# Cannabidiol Downregulates Myocardial de Novo Ceramide Synthesis Pathway in a Rat Model of High-Fat Diet-Induced Obesity

**DOI:** 10.3390/ijms23042232

**Published:** 2022-02-17

**Authors:** Tomasz Charytoniuk, Klaudia Sztolsztener, Patrycja Bielawiec, Adrian Chabowski, Karolina Konstantynowicz-Nowicka, Ewa Harasim-Symbor

**Affiliations:** Department of Physiology, Medical University of Bialystok, 15-222 Bialystok, Poland; t.charytoniuk@gmail.com (T.C.); klaudia.sztolsztener@umb.edu.pl (K.S.); patrycja.bielawiec@sd.umb.edu.pl (P.B.); adrian@umb.edu.pl (A.C.); karolina.konstantynowicz@umb.edu.pl (K.K.-N.)

**Keywords:** heart, sphingolipids, cannabidiol, fatty acid transporters, obesity

## Abstract

It is known that metabolic disturbances, including obesity, predispose to an increased incidence of cardiovascular diseases. Elevated consumption of dietary fat results in intramyocardial accumulation of lipids and their biologically active derivatives, which can disrupt the contractile function of the heart, its metabolism, and intracellular signaling pathways. Therefore, alternative methods, such as phytocannabinoids, are being sought for the treatment of obesity-related effects. In a model of rodent obesity (seven weeks of high-fat-diet (HFD) regime), we used cannabidiol—CBD therapy (intraperitoneal injections for 14 days; 10 mg/kg). High-performance and gas-liquid chromatographies were applied in order to determine sphingolipids in the heart and plasma as well as Western blotting for protein expression. Two-week CBD administration significantly inhibited the de novo ceramide synthesis pathway in the heart of HFD fed rats by lowering sphinganine and sphinganine-1-phosphate contents. The above reductions were accompanied by markedly diminished expressions of myocardial serine palmitoyltransferase 1 and 2 as well as ceramide synthase 5 and 6 in the HFD group with 2-week CBD treatment. To our knowledge, this research is the first that reveals unknown effects of CBD treatment on the heart, i.e., amelioration of de novo ceramide synthesis pathway in obese rats.

## 1. Introduction

Cardiovascular and metabolic diseases are one of the most common pathologies, whose number is rapidly increasing among different societies worldwide. Consequently, the prevalence of cardiometabolic disorders might be considered a global epidemic. It is known that continuous heart work is associated with increased cardiomyocytes metabolic demands and a wide range of substrates that can be utilized during ATP synthesis, e.g., fatty acids (FAs), carbohydrates (glucose and lactate), ketone bodies, and amino acids [1]. Although FAs and their β-oxidation constitute the prime source of cardiac energy (70–80%), glucose is also an essential molecule that, via its oxidation, provides 20–30% of the total ATP requirement in the myocardium [2,3]. Both in vitro and in vivo studies (models of high-fat diet), as well as clinical trials (models of obesity and metabolic syndrome), indicated that elevated availability of fatty acids may lead to congestive heart failure (CHF), hypertension, or cardiovascular diseases (CVDs) [4]. It is known that a higher intake of fat-rich food leads to increased circulating levels of long-chain fatty acids (LCFAs), supplying them directly to the coronary circulation. This promotes their intracellular influx to the cardiomyocytes, which can impair the physiological functioning of these cells. The Western pattern diet is remarkably rich in harmful saturated fatty acids (SFA) and trans fatty acids (TFA). This specific dietary consumption of high-fat food enhances uptake and storage of various lipid classes, e.g., sphingolipids and their essential fractions (i.e., ceramide—CER, sphingosine—SFO, and sphinganine—SFA), triacylglycerides (TAGs) as well as diacylglycerides (DAGs). It is known that these particular lipid species might be widely involved in the development of lipotoxicity, therefore metabolic disturbances in a cell as well [5]. Furthermore, a number of studies demonstrated an unequivocal lipotoxic effect of sphingolipids (e.g., elevated CER levels) on the myocardium and their contribution to heart failure, hypertension, and cardiometabolic pathologies development [6,7].

The endocannabinoid system (ECS) is a widespread lipid signaling system that participates in maintaining energy homeostasis, as well as numerous metabolic pathologies and CVDs [8,9]. Recently, an expanded endocannabinoid system has been defined as an endocannabinoidome (eCBome)—a system with more than 100 fatty acid-derived mediators and their receptors, as well as its anabolic and catabolic enzymes of more than 50 proteins [10]. One of the known molecules that widely interact with the eCBome is cannabidiol (CBD)—a non-psychoactive constituent of the Cannabis sativa plant [11]. A number of studies indicated that phytocannabinoids might be a promising therapeutic tool for alleviating the development of cardiometabolic-related pathologies, e.g., insulin resistance, inflammatory response, or lipotoxicity [12,13]. Although various CBD mechanisms of action were widely demonstrated in the myocardium, there are still few studies showing the relation between lipid metabolism and this phytocannabinoid in the cardiac muscle tissue [14,15]. We believe that our research, which presents the link between these aspects, may greatly contribute to the evaluation of novel therapeutic methods for CVDs with the usage of phytocannabinoids, which will be developed within the upcoming decade.

In order to achieve the above-mentioned objectives, we decided upon an experimental model of high-fat diet-induced obesity (7 weeks) in rats with concomitant injections of CBD (14 days). To show the influence of CBD on myocardial sphingolipid metabolism, we measured the contents of selected sphingolipid species (i.e., SFA, sphinganine-1-phosphate—SFA1P, CER, SFO, sphingosine-1-phosphate—S1P, and sphingomyelin—SM) in the left ventricle of the cardiac muscle and plasma. Moreover, we determined expressions of essential enzymes involved in the myocardial sphingolipid pathways together with fatty acid transporters expression, which reflects LCFAs availability within the cardiomyocytes.

## 2. Results

### 2.1. General Characteristic of the Experimental Model

**Figure 1:**
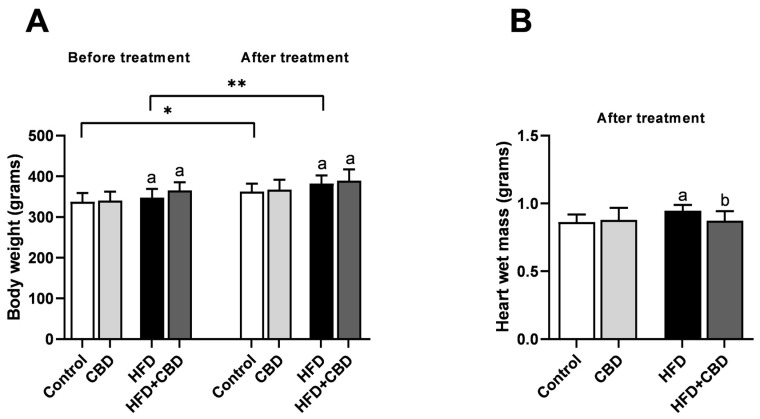
Body and heart weights parameters before and/or after 2-week cannabidiol (CBD) treatment in rats fed a standard diet (Control) or a high-fat diet (HFD). The results are expressed as mean values ± SD and are based on ten independent determinations in each experimental group. ^a^ *p* < 0.05 significant difference vs. respective control group, this refers to Figure 1A,B; ^b^ *p* < 0.05 significant difference vs. high-fat-fed group, this refers to Figure 1B; * *p* < 0.05 significant difference between control groups before and after CBD treatment ** *p* < 0.05 significant difference between high-fat-fed groups before and after CBD treatment.

Before 2-week CBD treatment, body weight of rats after five weeks of high-fat diet course was considerably elevated in both HFD and HFD+CBD groups compared to the respective control group (+3.08% and +8.32%, *p* < 0.05, Figure 1A, before treatment). Simultaneously, CBD administration did not substantially affect the body mass of the above-mentioned rats since their body weights were still increased (+5.32% and +7.25%, *p* < 0.05, Figure 1A, vs. control group, after treatment). Moreover, we observed significant alternations between two control groups as well as two HFD groups before and after 2-week CBD treatment (+7.63% and +9.95%, *p* < 0.05, Figure 1A, respectively). Nevertheless, in rats fed HFD and receiving CBD injections for 2 weeks, we observed a considerable decline in a wet mass of the heart (−7.81%, *p* < 0.05, Figure 1B, vs. HFD group alone), which was initially elevated (+9.76%, *p* < 0.05, Figure 1B, vs. control group).

### 2.2. Effects of 2-Week CBD Administration on the Plasma Sphinganine, Sphinganine-1-Phosphate, Ceramide, Sphingosine, and Sphingosine-1-Phosphate Contents in Both the Standard Chow and High-Fat Diet-Fed Rats

**Figure 2:**
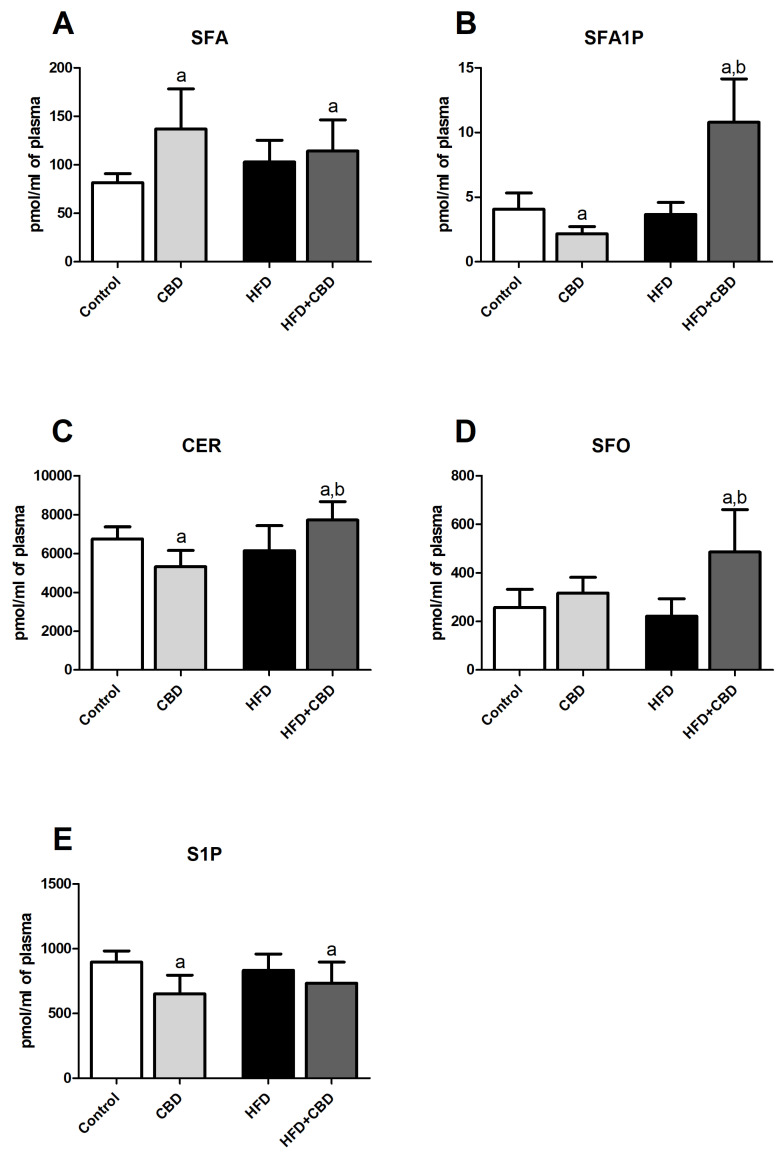
The content of plasma sphingolipids, i.e., sphinganine—SFA (**A**), sphinganine-1-phosphate—SFA1P (**B**), ceramide—CER (**C**), sphingosine—SFO (**D**), and sphingosine-1-phosphate—S1P (**E**) after 2-week cannabidiol (CBD) administration in the rats fed a standard diet (Control) or a high-fat diet (HFD). The components of sphingolipid pathway were measured by high-performance liquid chromatography (HPLC) method. The results are expressed as mean values ± SD and are based on ten independent determinations in each experimental group. ^a^ *p* < 0.05 significant difference vs. control group; ^b^ *p* < 0.05 significant difference vs. high-fat-fed group.

In the plasma, we noticed a considerable difference in the sphingolipid pathway components in response to CBD treatment during standard and high-fat feeding conditions. There was a substantial increase in SFA level in the CBD (+68.40%, *p* < 0.05, Figure 2A) and HFD+CBD (+40.37%, *p* < 0.05, Figure 2A) groups in relation to the control group. Moreover, 2-week cannabidiol administration caused a relevant reduction in the SFA1P concentration in rats fed a standard chow compared to the control group (−46.97%, *p* < 0.05, Figure 2B), with concomitant enhancement of this sphingolipid content in the rats fed a high-fat diet (+165.86%, *p* < 0.05, Figure 2B, vs. control group; +194.10%, *p* < 0.05, Figure 2B, vs. HFD group). Similar effects were observed in CER content in CBD group (−21.25%, *p* < 0.05, Figure 2C, vs. control group) and HFD+CBD group (+14.36%, *p* < 0.05, Figure 2C, vs. control group; +25.64%, *p* < 0.05, Figure 2C, vs. HFD group). Interestingly, 2-week CBD injections substantially increased the concentration of plasma SFO only in rats fed a high-fat chow (+88.15%, *p* < 0.05, Figure 2D, vs. control group; +118.50%, *p* < 0.05, Figure 2D, vs. HFD group). Simultaneously, in contrast to the control group, cannabidiol diminished plasma S1P fraction during both standard and high-fat feeding conditions (−27.44%, *p* < 0.05, Figure 2E; −18.01%, *p* < 0.05, Figure 2E, respectively).

### 2.3. Effects of 2-Week CBD Administration on the Total Myocardial Expression of Fatty Acid Transporters in Both the Standard Chow and High-Fat Diet-Fed Rats

**Figure 3:**
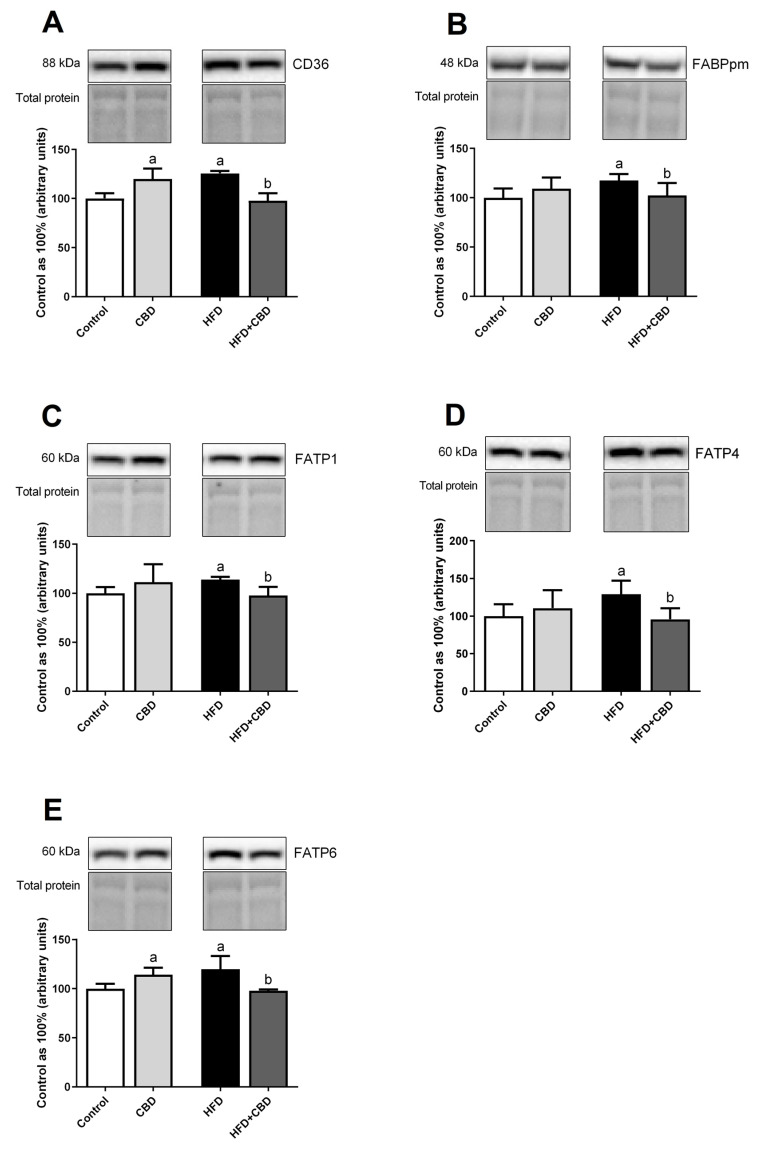
The total myocardial expression of fatty acid transport proteins, i.e., a cluster of differentiation/a scavenger receptor class B protein (SR-B2)—CD36 (**A**), plasma membrane fatty acid-binding protein—FABPpm (**B**), fatty acid transport protein 1—FATP1 (**C**), fatty acid transport protein 4—FATP4 (**D**), fatty acid transport protein 6—FATP6 (**E**) in the left ventricle after 2-week cannabidiol (CBD) administration in rats fed a standard diet (Control) or a high-fat diet (HFD). The protein expression was determined by Western blotting method, normalized to the total protein loading in each lane, and presented as a percentage difference in comparison to the control group, which was set at 100%. The results are expressed as mean values ± SD and are based on six independent determinations in each group. ^a^ *p* < 0.05 significant difference vs. control group; ^b^ *p* < 0.05 significant difference vs. high-fat feeding group.

All fatty acid transporters expressions in the left ventricle were detected at a significantly higher levels in animals fed HFD, i.e., a cluster of differentiation—CD36 (+25.54%, *p* < 0.05, Figure 3A), plasma membrane fatty acid binding protein—FABPpm (+17.61%, *p* < 0.05, Figure 3B), fatty acid transport protein 1—FATP1 (+13.99%, *p* < 0.05, Figure 3C), fatty acid transport protein 4—FATP4 (+29.28%, *p* < 0.05, Figure 3D), and fatty acid transport protein 6—FATP6 (+19.86%, *p* < 0.05, Figure 3E). Importantly, the aforementioned effects in the fatty acid overload conditions were abolished by the 2-week CBD injections, i.e., CD36 (−22.16%, *p* < 0.05, Figure 3A), FABPpm (−12.97%, *p* < 0.05, Figure 3B), FATP1 (−14.30%, *p* < 0.05, Figure 3C), FATP4 (−25.88%, *p* < 0.05, Figure 3D), and FATP6 (−18.41%, *p* < 0.05, Figure 3E) compared to the respective high-fat-diet group. Moreover, after a prolonged CBD treatment of the standard chow fed rats, we observed a significant increase only in the case of myocardial CD36 and FATP6 expressions (+19.81% and +14.35%, *p* < 0.05, Figure 3A,E, respectively) relative to the rats from appropriate control group.

### 2.4. Effects of 2-Week CBD Administration on the Myocardial Sphinganine, Sphinganine-1-Phosphate, Ceramide, Sphingosine, Sphingosine-1-Phosphate, and Sphingomyelin Contents in Both the Standard Chow and High-Fat Diet-Fed Rats

**Figure 4:**
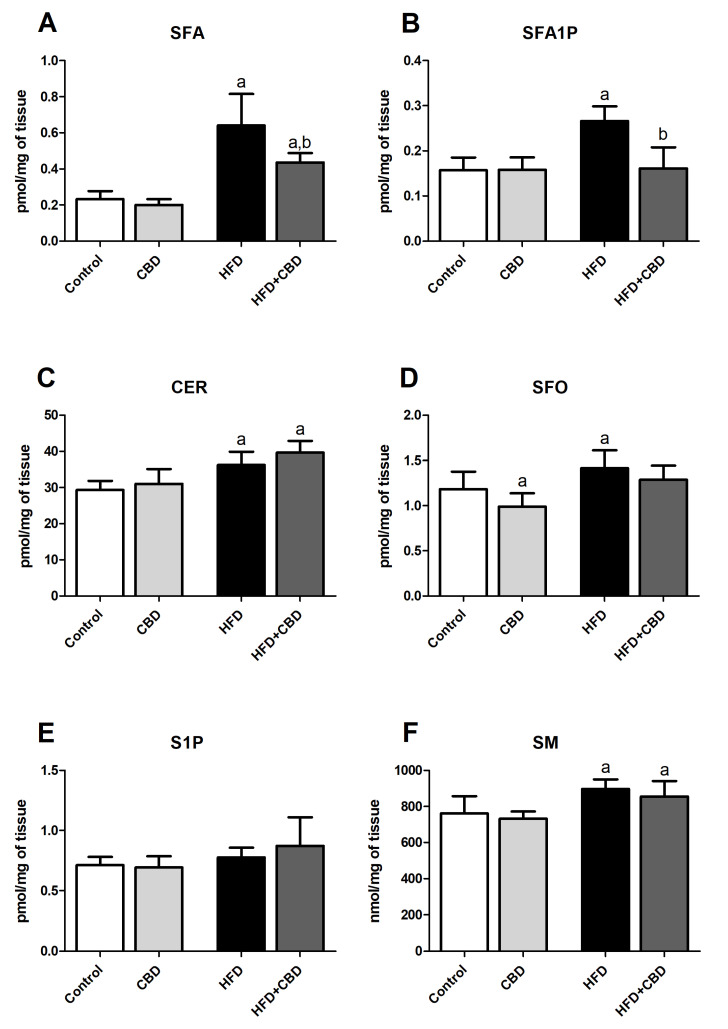
The content of myocardial sphingolipids, i.e., sphinganine—SFA (**A**), sphinganine-1-phosphate—SFA1P (**B**), ceramide—CER (**C**), sphingosine—SFO (**D**), sphingosine-1-phosphate—S1P (**E**), and sphingomyelin—SM (**F**) in the left ventricle tissue after 2-week cannabidiol (CBD) administration in rats fed a standard diet (Control) or a high-fat diet (HFD). The contents of sphingolipid pathway components were measured by high-performance liquid chromatography (HPLC) and gas-liquid chromatography (GLC) methods. The results are expressed as mean values ± SD and are based on ten independent determinations in each experimental group. ^a^ *p* < 0.05 significant difference vs. control group; ^b^ *p* < 0.05 significant difference vs. high-fat-fed group.

In the left ventricle of the cardiac muscle, we observed that a high-fat feeding intensified de novo sphingolipid synthesis due to the fact that in the HFD group there was a substantial increase in the level of SFA (+176.48%, *p* < 0.05, Figure 4A), SFA1P (+69.43%, *p* < 0.05, Figure 4B), CER (+23.67%, *p* < 0.05, Figure 4C), and SM (+17.75%, *p* < 0.05, Figure 4F) compared to the respective control group. Additionally, in comparison with the appropriate HFD group 2-week cannabidiol treatment, apart from SM content (*p* > 0.05, Figure 4F, vs. HFD group), markedly reduced the myocardial level of SFA (−32.18%, *p* < 0.05, Figure 4A) and SFA1P (−39.38%, *p* < 0.05, Figure 4B) in the HFD group. In contrast, prolonged CBD injections failed to decrease CER content (+35.13%, *p* < 0.05, Figure 4C) in the heart tissue of animals being on a high-fat diet compared to the rats fed standard chow. Given the changes of the left ventricle’s content of SFO, we detected a significant decrease in the SFO level after 2-week CBD treatment (−16.39%, *p* < 0.05, Figure 4D) and a concomitant increase in its concentration after excess lipid provision (+19.51%, *p* < 0.05, Figure 4D) compared to the control conditions. Moreover, a trend toward an increase induced by HFD or 2-week CBD treatment was found in S1P content (HFD: *p* = 0.0812 and CBD: *p* = 0.0760, Figure 4E) compared to the control group.

### 2.5. Effects of 2-Week CBD Administration on the Total Myocardial Expression of Enzymes Involved in the Sphingolipid Metabolism in Both the Standard Chow and High-Fat Diet-Fed Rats

**Figure 5:**
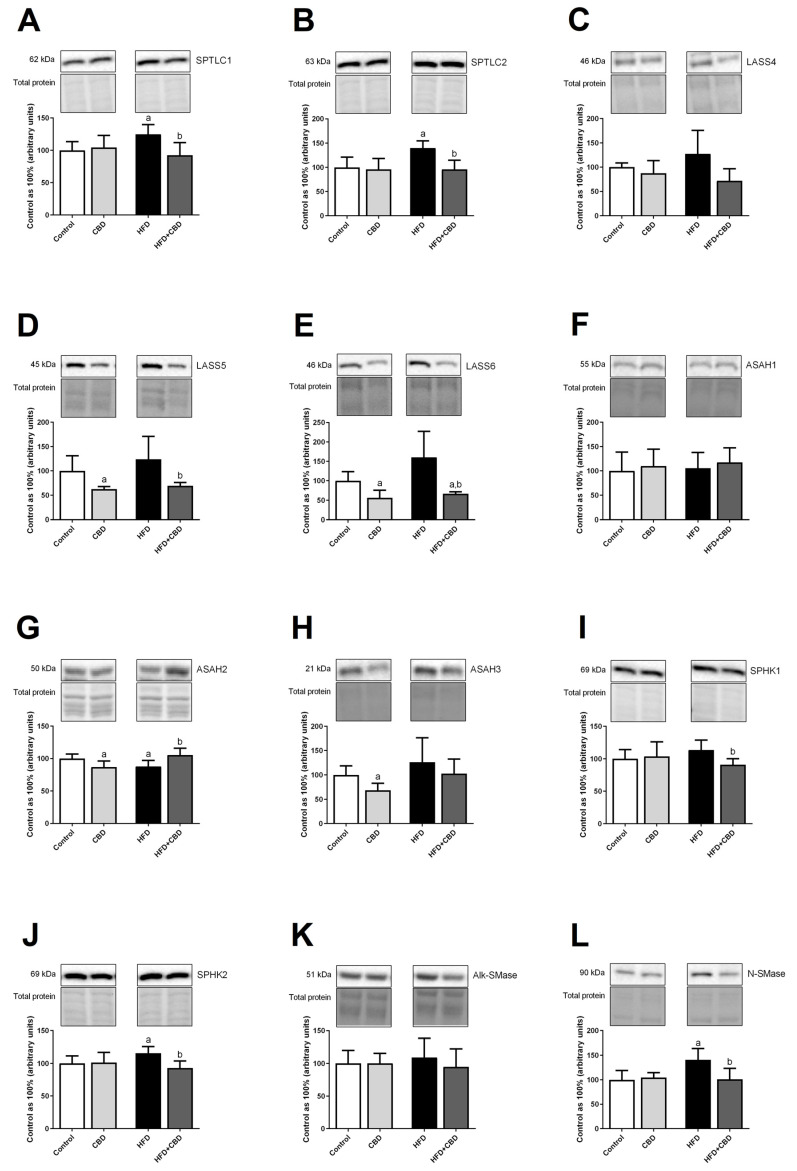
The total myocardial expression of the enzymes involved in the sphingolipid metabolism, i.e., serine palmitoyltransferase, long chain base subunit 1—SPTLC1 (**A**), serine palmitoyltransferase, long chain base subunit 2—SPTLC2 (**B**), ceramide synthase 4—LASS4 (**C**), ceramide synthase 5—LASS5 (**D**), ceramide synthase 6—LASS6 (**E**), acid ceramidase—ASAH1 (**F**), neutral ceramidase—ASAH2 (**G**), alkaline ceramidase—ASAH3 (**H**), sphingosine kinase 1—SPHK1 (**I**), sphingosine kinase 2—SPHK2 (**J**), alkaline sphingomyelinase—Alk-SMase) (**K**), and neutral sphingomyelinase—N-SMase (**L**) in the left ventricle homogenates after 2-week cannabidiol (CBD) administration in rats fed a standard diet (Control) or a high-fat diet (HFD). The protein expression was determined by Western blotting method, normalized to the total protein loading in each lane, and presented as a percentage difference in comparison to the control group, which was set at 100%. The results are expressed as mean values ± SD and are based on six independent determinations in each group. ^a^ *p* < 0.05 significant difference vs. control group; ^b^ *p* < 0.05 significant difference vs. high-fat feeding group.

As expected, a high-fat feeding caused a significant increase in the total expression of serine palmitoyltransferase 1 (SPTLC1) and serine palmitoyltransferase 2 (SPTLC2) in the left ventricle tissue homogenates (+24.70%, *p* < 0.05, Figure 5A; +39.77%, *p* < 0.05, Figure 5B, respectively) compared to the control rats. Importantly, CBD treatment considerably diminished the expression of the aforementioned de novo ceramide synthesis pathway enzymes, i.e., SPTLC1 (−25.85%, *p* < 0.05, Figure 5A) and SPTLC2 (−31.50%, *p* < 0.05, Figure 5B) in high-fat diet-fed rats in relation to the rats from HFD group alone. Similarly, the total myocardial expression of ceramide synthase 5 (LASS5) and ceramide synthase 6 (LASS6) revealed a significant decrease in the CBD group alone (−37.37% and −44.05%, *p* < 0.05, Figure 5D,E vs. control group, respectively) as well as the rats receiving high-fat diet and CBD (−44.02% and −58.51%, *p* < 0.05, Figure 5D,E vs. HFD group, respectively). In the case of ceramidases expression in the salvage ceramide synthesis pathway, we did not observe any significant changes in the myocardial expression of acid ceramidase (ASAH1) (*p* > 0.05, Figure 5F) in both standard and high-fat diet-fed rats. On the other hand, the total myocardial expression of neutral ceramidase (ASAH2) and alkaline ceramidase (ASAH3) was considerably reduced after 2-week CBD injections during standard conditions (−13.18%, *p* < 0.05, Figure 5G and −31.71%, *p* < 0.05, Figure 5H, respectively). Furthermore, obesity induced by a high-fat feeding provoked a crucial decrease in ASAH2 expression (−12.19%, *p* < 0.05, Figure 5G, vs. control group), which was further elevated by prolonged CBD treatment (+20.03%, *p* < 0.05, Figure 5G) in comparison to the rats from the HFD group. After the HFD course, an increase in the myocardial expression was observed in the case of both sphingosine kinase 1—SPHK1 (*p* > 0.05, Figure 5I) and sphingosine kinase 2—SPHK2 (+15.65%, *p* < 0.05, Figure 5H); however, only the change in the expression of SPHK2 was significant. Application of CBD also noticeably lowered both SPHK1 and SPHK2 expressions in the left ventricle after enhanced lipid provision (−21.25%, *p* < 0.05, Figure 5I; −19.79%, *p* < 0.05, Figure 5J, respectively) in relation to the appropriate HFD group. In all the examined groups, the total myocardial expression of alkaline sphingomyelinase (Alk-SMase) remained unchanged (*p* > 0.05, Figure 5K), whereas a substantial increase in the neutral sphingomyelinase (N-SMase) level after a high-fat chow administration (+40.75%, *p* < 0.05, Figure 5L) compared to the control conditions was noticed and was further reduced by CBD injections (−24.01%, *p* < 0.05, Figure 5L, vs. HFD group).

### 2.6. Effects of 2-Week CBD Administration on the Total Myocardial Expression of the Endocannabinoid System Components in Both the Standard Chow and High-Fat Diet-Fed Rats

**Figure 6:**
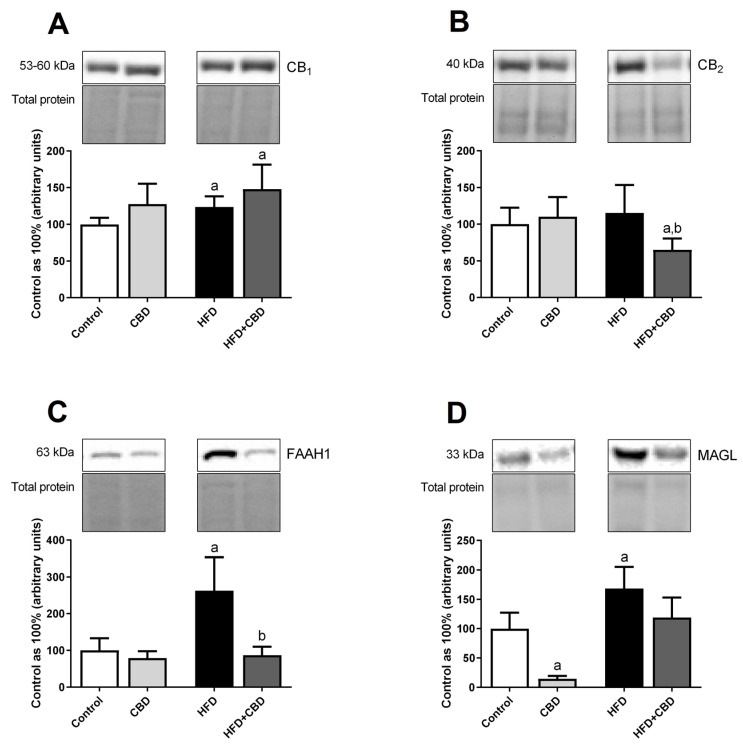
The total myocardial expression of cannabinoid receptor 1 (CB_1_) (**A**), cannabinoid receptor 2 (CB_2_) (**B**), fatty acid amide hydrolase 1 (FAAH1) (**C**), and monoacylglycerol lipase (MAGL) (**D**) in the left ventricle homogenates after 2-week cannabidiol (CBD) administration in rats fed a standard diet (Control) or a high-fat diet (HFD). The protein expression was determined by Western blotting method, normalized to the total protein loading in each lane, and presented as a percentage difference in comparison to the control group, which was set at 100%. The results are expressed as mean values ± SD and are based on six independent determinations in each group. ^a^ *p* < 0.05 significant difference vs. control group; ^b^ *p* < 0.05 significant difference vs. high-fat feeding group.

In the left ventricle tissue, a high-fat chow administration substantially increased the total expression of cannabinoid receptor 1 (CB_1_) compared to the standard conditions (+21.69%, *p* < 0.05, Figure 6A), whereas 2-week CBD administration had an additive effect on this alternation (+48.17%, *p* < 0.05, Figure 6A, vs. control group). Simultaneously, after CBD treatment, we observed a substantial reduction in cannabinoid receptor 2 (CB_2_) expression in the lipid overload conditions in relation to the untreated control and HFD groups (−35.13% and −43.80%, *p* < 0.05, Figure 6B, respectively). After high-fat feeding, a substantial elevation in the myocardial fatty acid amide hydrolase 1 (FAAH1) (+162.52%, *p* < 0.05, Figure 6C) and monoacylglycerol lipase (MAGL) (+64.48%, *p* < 0.05, Figure 6D) expressions compared to the standard-chow-fed control rats was observed. Most importantly, 2-week CBD treatment considerably reduced the above-mentioned effect in the case of FAAH1 expression (−66.87%, *p* < 0.05, Figure 6C) in comparison to the HFD group. Moreover, 2-week CBD injections significantly diminished the expression of MAGL in the rats fed standard chow (−85.51%, *p* < 0.05, Figure 6D, vs. control group).

## 3. Discussion

**Scheme 1:**
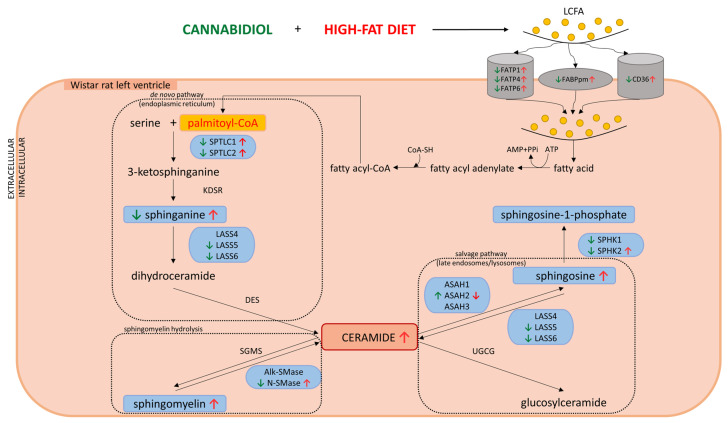
Impact of high-fat diet (HFD) and 2-week cannabidiol (CBD) administration on the sphingolipid metabolic pathway in rat’s cardiomyocytes. ↑, an increase; ↓, a decrease; red arrows represent high-fat feeding for 7 weeks; green arrows represent the effects of 2-week CBD treatment (the last two weeks of high-fat diet regime); long chain fatty acid (LCFA), cluster of differentiation/a scavenger receptor class B protein (SR-B2) (CD36), plasma membrane fatty acid binding protein (FABPpm), fatty acid transport protein 1 (FATP1), fatty acid transport protein 4 (FATP4), fatty acid transport protein 6 (FATP6), serine palmitoyltransferase, long chain base subunit 1 (SPTLC1), serine palmitoyltransferase, long chain base subunit 2 (SPTLC2), ceramide synthase 4 (LASS4), ceramide synthase 5 (LASS5), ceramide synthase 6 (LASS6), dihydroceramide desaturase (DES), acid ceramidase (ASAH1), neutral ceramidase (ASAH2), alkaline ceramidase (ASAH3), sphingosine kinase 1 (SPHK1), sphingosine kinase 2 (SPHK2), sphingomyelin synthase (SGMS), alkaline sphingomyelinase (Alk-SMase), neutral sphingomyelinase (N-SMase), and UDP-glucose ceramide glucosyltransferase (UGCG).

For the first time, our study has shown the relationship between myocardial sphingolipid metabolism and CBD treatment in high-fat diet-induced obesity in rats. We found that this phytocannabinoid in such conditions is a modulator of the de novo ceramide synthesis pathway along with the salvage route, however, to a different extent in the plasma and cardiac muscle. We noticed a remarkable decrease in both SFA and SFA1P myocardial contents after CBD treatment during a high-fat feeding regime (Scheme 1) [16]. Possibly due to the extracellular sphingolipid efflux, e.g., from the liver, significantly increased concentrations of these two sphingolipids were observed in Wistar rats’ blood plasma. We believe that this might be considered as a cell protection mechanism against ceramide synthesis since SFA is a primary component of the ceramide de novo synthesis pathway. Three different metabolic pathways of CER generation are defined: (1) de novo synthesis pathway via serine palmitoyltransferase and sphinganine formation, (2) sphingomyelin hydrolysis via sphingomyelinase, and (3) the salvage pathway—a synthesis of ceramide from sphingosine via ceramide synthase (Scheme 1). Although our study did not demonstrate significant differences in CER content in the cardiac tissue after 2-week CBD treatment in a high-fat-diet group in comparison with HFD alone, we noticed a considerable elevation in blood plasma CER concentration in the same experimental group. An extracellular rise of CER content might be correlated with its enhanced cell efflux, for instance, from the liver, to reduce the intracellular CER accumulation, especially when there is FAs oversupply [17]. This sphingolipid, when accumulated, is known to demonstrate numerous destructive cell effects, including the development of metabolic disorders such as impairments of insulin signaling pathway and related insulin resistance [18,19]. Parra et al. demonstrated that CER affecting mitochondrial dynamics might be involved in the apoptosis of cardiomyocytes [20]. With regard to the above-mentioned matter, Law et al. showed that very-long-chain ceramides can develop lipotoxic effects, e.g., mitochondrial dysfunction, oxidative stress, and cell apoptosis in cardiomyocytes [21]. Interestingly, a study conducted by Bielawska et al. demonstrated that CER affects signal transduction leading to cardiomyocytes’ apoptosis during heart ischemia and reperfusion injury [22]. Considering the enzymes involved in the sphingolipid pathway, we observed a remarkable decrease in the expression of enzymes controlling the de novo ceramide synthesis pathway, namely SPTLC1 and 2, as well as LASS5 and 6 after 2-week CBD treatment in HFD fed Wistar rats, which is in line with the above-mentioned downregulation. Our study also indicated that CBD treatment in high-fat feeding lowered the expression of proteins from the salvage pathway, i.e., SPHK1 and 2, with simultaneous augmentation of the expression of neutral ceramidase in comparison with the high-fat diet-fed rats (Scheme 1). The above-mentioned alterations in the myocardial expression of enzymes are in accordance with the decreased intracellular concentration of two sphingolipid fractions, which are ceramide precursors—SFA in the de novo synthesis pathway and SFO in the salvage pathway (a trend toward reduction). This might lead to the presumption that CBD in conditions of increased FAs consumption inhibits two different ceramide synthesis pathways. We are aware of the fact that this should be confirmed in isolated primary cardiomyocytes with the additional presence of a specific inhibitor (e.g., myriocin—a serine palmitoyltransferase inhibitor); therefore, it can be considered as a limitation of this study. Nonetheless, we do believe that the obtained data undoubtedly revealed a causal relationship between downregulation of the de novo ceramide synthesis pathway and 2-week CBD administration in FAs oversupplied rats, which in the long-term may prevent negative consequences of CER accumulation in the heart. Although we did not observe a significant difference in myocardial tissue S1P content between examined groups, there was a trend toward its enhancement in the HFD+CBD group, whereas plasma concentration of this sphingolipid derivative was reduced after CBD introduction irrespective of applied diet type. Some researchers emphasize that signaling of S1P may exhibit both anti- and pro-fibrotic effects in cardiac fibrosis [23]. Furthermore, studies conducted on mice demonstrated that S1PR1 (sphingosine-1-phosphate receptor 1) signaling might be considered as a novel therapeutic option for hypertension via vascular relaxation [24]. Other studies carried out by Jin et al. showed that S1P exhibits a cardioprotective role since it protects the ex vivo heart from ischemia–reperfusion injury [25]. It corresponds with the recent study revealing vasoprotective effects of chronic CBD treatment in hypertensive rats, such as a reduction of hypertrophy and improvement of the endothelium-dependent vasodilation [26]. Simultaneously, we have shown in the previous research that chronic administration of CBD (10 mg/kg) was not successful in reducing both blood pressure value and heart rate in an animal model of primary and secondary hypertension (spontaneously and deoxycorticosterone hypertensive rats) [27]. Taking into account the above and present data, it is probable that CBD indirectly, through alternations in sphingolipid metabolism, triggers vaso- and cardioprotective outcomes. Considering the concentration of sphingomyelin, we observed a trend toward a decrease in its content in the high-fat-diet group exposed to CBD, which means that CBD probably leads to an activation of the SM breakdown. Our data are consistent with studies conducted by Burstein et al., who evaluated the effect of CBD on SM content in fibroblasts and demonstrated a considerable decrease in SM concentration after exposure to CBD treatment [28]. It is known that sphingomyelinases are widely related to cardiovascular diseases [29]. Although our Western blot analysis after treatment with CBD indicated decreased expression of both enzymes regulating the metabolism of SM—alkaline and neutral sphingomyelinase, where a significant difference in comparison to the HFD group was observed in N-SMase expression. Interestingly, Klevstig et al. indicated that inhibition of acid sphingomyelinase might result in the reduction of CER accumulation in the post-ischemic cardiac tissue. However, the same study also revealed that inhibition of SMase in mice did not improve heart function after induced myocardial infarction despite decreasing CER storage [30]. Moreover, a study conducted by Hernandez et al. on rats cardiac myocytes indicated that neutral SMase and the subsequent signaling of CER might be one of the earliest cardiac tissue responses to oxidative stress in a model of myocardial ischemia [31].

It is known that a higher intake of fat-rich food leads to increased circulating levels of LCFAs in the coronary circulation, which promotes their uptake across the plasma membrane to the cardiomyocytes. Three membrane-associated fatty acid protein transporters are known to greatly facilitate LCFAs uptake in cardiomyocytes (up to 80% of the total FAs transmembrane transport), i.e., CD36, FABPpm, and FATP1, 4, and 6 [32]. A number of animal studies and human clinical trials based on models of obesity or type 2 diabetes mellitus revealed that accumulation of different lipid fractions, including CER, is correlated with an increased expression of these protein transporters, mainly CD36 and FABPpm, in metabolically active tissues, i.e., the heart, adipose tissue, skeletal muscles, and liver [33,34]. To our knowledge, this study is the first in which it is demonstrated a remarkable decline in the expression of all five examined transporters, i.e., CD36, FABPpm, FATP1, 4, and 6, after 2-week CBD treatment under conditions of high-fat diet-induced obesity in Wistar rats (Scheme 1). Therefore, based on the above data, we can assume that CBD via its influence on LCFA transporters possibly leads to a diminished accumulation of lipids and their derivatives in the cardiomyocytes also through the de novo CER synthesis pathway, which is consistent with lower heart mass in HFD fed rats after CBD treatment even though the total body mass was not altered. This, in turn, can be considered as a cardioprotective mechanism during increased dietary fatty acid intake. However, the exact mechanism by which CBD decreases the total myocardial expression of LCFAs protein transporters should also be examined in the in vitro model. One of the hypotheses, which should be further investigated, includes peroxisome proliferator-activated receptors (PPARα and PPARβ/δ) since they serve as a link between LCFA protein transporters and the ECS [35,36]. Nevertheless, it should be underlined that in our research, a reduced expression of fatty acid transporters (a protective mechanism against lipotoxicity) is closely related with downregulated de novo ceramide synthesis pathway in the cardiac muscle of obese rats as the consequence of lower LCFAs influx. Our observations are in line with other studies in which CBD treatment resulted in a significant reduction of intracellular lipid droplets (e.g., TAGs fraction) as well as lipogenesis in hepatocytes [37].

The expanded endocannabinoid system includes elementarily G-protein-coupled receptors, known as CB_1_ and CB_2_, which mediate the effects of cannabinoids. The CB_1_ receptor is principally expressed in the central nervous system, whereas CB_2_ is the predominant cannabinoid receptor within the immune system’s cells [38]. Cannabidiol is known to interact with various complex signaling systems involved in eCBome; however, it has a very low affinity to cannabinoid receptors [39]. We can distinguish two endocannabinoids (eCBs) that are endogenous agonists of CB_1_ and CB_2_ receptors—anandamide (AEA) and 2-arachidonoylglycerol (2-AG), [40]. Our study indicated a significant decrease in the myocardial CB_2_ receptor expression in the HFD+CBD group in comparison with both control and HFD groups. This downregulatory effect of the CBD treatment on CB_2_ receptor expression was causally related with lowered expression of both fatty acid amide hydrolase (FAAH) and monoacylglycerol lipase (MAGL; a trend toward a decrease) in the HFD group after 2-week CBD application. Since FAAH catalyzes the hydrolysis of AEA to arachidonic acid and ethanolamine [41], its inhibition by CBD in high-fat diet-fed rats probably resulted in an increased level of eCBs, which in turn provoked downregulation of the myocardial expression of the CB_2_ receptor. Even though CBD was successful in diminishing FAAH expression, it did not affect augmented by high-fat-diet expression of CB_1_, presumably due to the fact that CBD itself is known to be a negative allosteric modulator of this cannabinoid receptor. Additionally, FAAH, apart from AEA and 2-AG, degrades endocannabinoid-like molecules, namely N-oleoylethanolamide (OEA) and N-palmitoylethanolamide (PEA) [42]. It was shown that OEA binds PPARα (with high affinity) and PPARβ/δ, whereas PEA is an agonist of only PPARα. Interestingly, Fu et al. displayed that OEA triggers satiety and reduces body weight gain in wild-type mice, but not in PPARα knockout animals [43]. Moreover, PPARα and β/δ are regulators of lipid metabolism, also through modulation of the expression of LCFAs protein transporters (e.g., CD36, FABPpm, or FATP1) in different tissues, including cardiac muscle [36,43]. Since in our study, we observed a considerable decline in the FAAH expression in the HFD+CBD group (vs. HFD-fed rats), it is probable that via OEA and/or PEA PPARs activation was affected, which in turn produced changes in de novo CER synthesis pathway and the expression of LCFAs protein transporters. Therefore, we can hypothesize that PPARs link sphingolipid metabolism, the ECS, and fatty acid transporters, which should be confirmed in in vitro experiments. Moreover, endocannabinoids are considered to be very promising cardioprotective molecules. A number of studies indicated their significant role in both physiological conditions [44] and various cardiac pathologies, e.g., myocardial infarction, hypertrophy, or hypertension affecting inflammatory response, infarct size, or blood pressure value [13,45]. A study conducted by Bátkai et al. demonstrated on FAAH knockout mice a reduced inflammation, oxidative/nitrative stress, and cardiomyocytes apoptosis in age-related cardiac dysfunctions, compared to the wild types of mice [46].

## 4. Materials and Methods

### 4.1. Animals and Experimental Protocol

The experiment was conducted on male Wistar rats (70–100 g) purchased from the Center for Experimental Medicine of the Medical University of Bialystok, Poland. The rats were housed in standard cages under controlled animal holding conditions (22 ± 2 °C with a reverse light-dark cycle of 12 h/12 h) with free access to drinking water and commercial laboratory chow (Labofeed B, Animal Feed Manufacturer “Morawski”, Kcynia, Poland). The protocol of the study was approved by the Animal Ethics Committee in Olsztyn (No. 71/2018). After one week period of acclimatization, the animals were assigned to four groups: (1) control group—rats fed a standard diet (kcal distribution: 12.4% of energy from fat, 57.1% from carbohydrates, and 30.5% from protein), (2) CBD group—rats fed a standard diet and treated with CBD, (3) HFD group—rats fed a high-fat diet (kcal distribution: 60% of energy from fat, 20% from carbohydrates, and 20% from protein), and (4) HFD+CBD group—rats fed a high-fat diet and treated with CBD. Each experimental group included 10 rats, and the total duration of feeding rats with the standard laboratory chow or high-fat diet was seven weeks. Starting from the fifth week of a diet regime, rats were injected with CBD or its vehicle for the consecutive 14 days of the experiment. The animals from both standard diet and HFD-fed groups received intraperitoneal (i.p.) injections of synthetic CBD (purity: ≥99%; THC Pharm GmbH, Frankfurt, Germany) in a dose of 10 mg/kg of body mass (3:1:16, ethanol, Tween-80, and 0.9% NaCl) [15]. Simultaneously corresponding control and HFD groups were administered with the vehicle once a day for 14 days. Body mass was monitored throughout the whole experiment. Briefly, 24 h after the last injection of CBD or its vehicle, rats were anesthetized by i.p. injections of pentobarbital (80 mg/kg of body weight). Then, blood samples were collected into heparinized tubes through the inferior vena cava and centrifuged, and plasma was separated. The heart was excised, visible fatty tissue was removed, then weighted, and samples of the cardiac muscle were taken (i.e., the left ventricle). Thereafter, collected samples were at once immediately frozen in liquid nitrogen using precooled aluminum tongs and stored until further analysis at −80 °C.

### 4.2. Plasma and Heart Tissue Lipid Analysis

In the left ventricle and plasma samples, we measured ceramide, sphinganine, sphinganine-1-phosphate, sphingosine, and sphingosine-1-phosphate contents by means of high-performance liquid chromatography (HPLC), as previously described [47]. In brief, the heart tissue was homogenized, and lipids were extracted into the chloroform phase. The new tubes containing 40 pmol of N-palmitoyl-D-erythro-sphingosine (C17 base) as an internal standard were prepared, and lipid extracts aliquots were transferred to them. Thereafter, the alkaline water was added to the samples to form deacylate ceramide. The lipid residues (free sphinganine and sphingosine) released from ceramide were converted to their o-phthalaldehyde derivatives and assayed using the HPLC system (PROSTAR; Varian Inc., Palo Alto, CA, USA) equipped with a fluorescence detector and C18 reversed-phase column (Varian Inc. OmniSpher 5, 4.6 × 150 mm).

The intramyocardial content of sphingomyelin in the left ventricle was estimated by means of gas-liquid chromatography (GLC). Briefly, cardiac muscle samples were powdered, and lipids were extracted according to the Folch method in a chloroform–methanol (2:1 vol/vol) solution [48]. Then, the SM fraction was separated by thin-layer chromatography (TLC) on silica gel plates (Silica Plate 60, 0.25 mm; Merck, Darmstadt, Germany). Subsequently, individual fatty acid methyl esters were identified and quantified based on the standards retention times with the use of the GLC (Hewlett-Packard 5890 Series II gas chromatograph, HP-INNOWax capillary column). The total amount of SM was calculated as the sum of the particular fatty acid species content in the selected fraction and expressed in nanomoles per gram of wet tissue.

### 4.3. Western Blotting

A routine Western blotting procedure was used to determine protein expression, as it was described in detail previously [49]. In brief, the left ventricle samples were homogenized in an ice-cold radioimmunoprecipitation assay (RIPA) buffer with the addition of protease and phosphatase inhibitors (Roche Diagnostics GmbH, Manheim, Germany). The total protein concentration in the homogenates was ascertained using the bicinchoninic acid (BCA) protein assay kit with bovine serum albumin (BSA) as a standard. Thereafter, proteins (30 μg) were electrophoretically separated on CriterionTM TGX Stain-Free Precast Gels (Bio-Rad, Hercules, CA, USA) and then transferred onto polyvinylidene fluoride (PVDF) or nitrocellulose membranes, depending on the transfer method. The membranes, after blocking in Tris-buffered saline with Tween-20 (TBST) with 5% non-fat dry milk or BSA, were incubated overnight at 4 °C with selected primary antibodies, i.e., SPTLC1 (1:500; Abcam, Cambridge, UK), SPTLC2 (1:500; Santa Cruz Biotechnology, Inc., Dallas, TX, USA), SPHK1 (1:500; Sigma Aldrich, Saint Louis, MO, USA), SPHK2 (1:500; Sigma Aldrich, Saint Louis, MO, USA), ceramide synthase 4 (LASS4, 1:1000; Sigma Aldrich, Saint Louis, MO, USA), LASS5 (1:500; Santa Cruz Biotechnology, Inc., Dallas, TX, USA), LASS6 (1:500; Abcam, Cambridge, UK), ASAH1 (1:200; Santa Cruz Biotechnology, Inc., Dallas, TX, USA), ASAH2 (1:200; Santa Cruz Biotechnology, Inc., Dallas, TX, USA), ASAH3 (1:1000; Sigma Aldrich, Saint Louis, MO, USA), Alk-SMase (1:500; Santa Cruz Biotechnology, Inc., Dallas, TX, USA), N-SMase (1:500; Santa Cruz Biotechnology, Inc., Dallas, TX, USA), CD36 (1:500; Santa Cruz Biotechnology, Inc., Dallas, TX, USA), FABPpm (1:8000; Abcam, Cambridge, UK), FATP (1:500; Santa Cruz Biotechnology, Inc., Dallas, TX, USA), FATP4 (1:1000; Abcam, Cambridge, UK), and FATP6 (2 µg/mL, ThermoFisher Scientific, USA), CB_1_ (1:500; Abcam, UK), CB_2_ (1:500; Abcam, UK), FAAH1 (1:500; Abcam, Cambridge, UK), and MAGL (1:500; Santa Cruz Biotechnology, Inc., Dallas, TX, USA). To detect proteins, the membranes were washed and subsequently incubated with an appropriate secondary antibody (1:3000, Santa Cruz Inc., Dallas, TX, USA) conjugated with horseradish peroxidase (HRP). After the visualization with chemiluminescence substrate (Clarity Western ECL Substrate; Bio-Rad, Hercules, CA, USA), the obtained protein bands were quantified densitometrically with a ChemiDoc visualization system (Image Laboratory Software Version 6.0.1; Bio-Rad, Warsaw, Poland). The total expression of target proteins was quantified with stain-free gels using the total protein normalization method (Bio-Rad) [50]. Briefly, the chemiluminescent blot channel intensity values were adjusted for variation in the protein loading between different lanes. Thus, the total protein normalization included comparing the chemiluminescence signal in each lane to its corresponding stain-free lane. The data are shown as the percentage of the control group, which was set as 100%, and are based on six independent determinations.

### 4.4. Statistical Analysis

All data are expressed as mean values ± SD or percentage of the control group based on ten (HPLC and GLC) or six (Western blotting) independent determinations. Obtained data were subjected to the Shapiro–Wilk test and Barlett’s test to assess the distribution of values and homogeneity of the variance. Statistical differences between groups were examined by one-way test ANOVA followed by an appropriate post hoc test using GraphPad Prism version 7.0 for Windows (GraphPad Software, La Jolla, CA, USA). Results were considered to be statistically significant at *p* < 0.05.

## 5. Conclusions

Our experimental model has, for the first time, revealed the influence of 2-week CBD treatment on the myocardial and plasma sphingolipid metabolism in a rat model of obesity. We have shown that CBD application is an effective tool in attenuating de novo ceramide synthesis pathway, mainly by decreasing SFA and SFA1P contents as well as SPTLC1, 2 and LASS5, 6 expressions in the left ventricle of rats with increased dietary fatty acid intake. Those alternations correspond with a reduced total myocardial expression of fatty acid transporters in such conditions (Scheme 1). Even though CBD significantly inhibited de novo ceramide synthesis in high-fat diet-fed rats, its myocardial content was still elevated, probably due to alterations in the salvage pathway. Moreover, based on our research, we propose, in a future perspective, to examine whether PPARs are the link between phytocannabinoids, sphingolipid metabolism, and fatty acid protein transporters. Taken altogether, it seems that the major action of CBD in fatty acids oversupplied conditions is related to limitation in LCFA influx and following formation of intracellular sphingolipid derivatives, which appears to be an attractive therapeutic strategy in obesity.

## Data Availability

Not applicable.

## References

[1] Karwi Q.G., Uddin G.M., Ho K.L., Lopaschuk G.D. (2018). Loss of Metabolic Flexibility in the Failing Heart. Front. Cardiovasc. Med..

[2] Chabowski A., Górski J., Glatz J.F., Luiken J.J., Bonen A. (2008). Protein-mediated Fatty Acid Uptake in the Heart. Curr. Cardiol. Rev..

[3] Lopaschuk G.D., Ussher J.R., Folmes C.D., Jaswal J.S., Stanley W.C. (2010). Myocardial fatty acid metabolism in health and disease. Physiol. Rev..

[4] Briggs M.A., Petersen K.S., Kris-Etherton P.M. (2017). Saturated Fatty Acids and Cardiovascular Disease: Replacements for Saturated Fat to Reduce Cardiovascular Risk. Healthcare.

[5] Bandet C.L., Tan-Chen S., Bourron O., Le Stunff H., Hajduch E. (2019). Sphingolipid Metabolism: New Insight into Ceramide-Induced Lipotoxicity in Muscle Cells. Int. J. Mol. Sci..

[6] Park T.S., Goldberg I.J. (2012). Sphingolipids, lipotoxic cardiomyopathy, and cardiac failure. Heart Fail. Clin..

[7] Spijkers L.J., van den Akker R.F., Janssen B.J., Debets J.J., De Mey J.G., Stroes E.S., van den Born B.J., Wijesinghe D.S., Chalfant C.E., MacAleese L. (2011). Hypertension is associated with marked alterations in sphingolipid biology: A potential role for ceramide. PLoS ONE.

[8] Pacher P., Steffens S. (2009). The emerging role of the endocannabinoid system in cardiovascular disease. Semin. Immunopathol..

[9] Kunos G., Osei-Hyiaman D., Liu J., Godlewski G., Bátkai S. (2008). Endocannabinoids and the control of energy homeostasis. J. Biol. Chem..

[10] Di Marzo V., Silvestri C. (2019). Lifestyle and Metabolic Syndrome: Contribution of the Endocannabinoidome. Nutrients.

[11] Chye Y., Christensen E., Solowij N., Yücel M. (2019). The Endocannabinoid System and Cannabidiol’s Promise for the Treatment of Substance Use Disorder. Front. Psychiatry.

[12] Di Marzo V., Piscitelli F., Mechoulam R. (2011). Cannabinoids and endocannabinoids in metabolic disorders with focus on diabetes. Handb. Exp. Pharmacol..

[13] Eid B.G. (2018). Cannabinoids for Treating Cardiovascular Disorders: Putting Together a Complex Puzzle. J. Microsc. Ultrastruct..

[14] Durst R., Danenberg H., Gallily R., Mechoulam R., Meir K., Grad E., Beeri R., Pugatsch T., Tarsish E., Lotan C. (2007). Cannabidiol, a nonpsychoactive Cannabis constituent, protects against myocardial ischemic reperfusion injury. Am. J. Physiol. Heart Circ. Physiol..

[15] Rajesh M., Mukhopadhyay P., Bátkai S., Patel V., Saito K., Matsumoto S., Kashiwaya Y., Horváth B., Mukhopadhyay B., Becker L. (2010). Cannabidiol attenuates cardiac dysfunction, oxidative stress, fibrosis, and inflammatory and cell death signaling pathways in diabetic cardiomyopathy. J. Am. Coll. Cardiol..

[16] Kitatani K., Idkowiak-Baldys J., Hannun Y.A. (2008). The sphingolipid salvage pathway in ceramide metabolism and signaling. Cell Signal.

[17] Watt M.J., Barnett A.C., Bruce C.R., Schenk S., Horowitz J.F., Hoy A.J. (2012). Regulation of plasma ceramide levels with fatty acid oversupply: Evidence that the liver detects and secretes de novo synthesised ceramide. Diabetologia.

[18] Kuzmenko D.I., Klimentyeva T.K. (2016). Role of Ceramide in Apoptosis and Development of Insulin Resistance. Biochemistry.

[19] Aburasayn H., Al Batran R., Ussher J.R. (2016). Targeting ceramide metabolism in obesity. Am. J. Physiol. Endocrinol. Metab..

[20] Parra V., Eisner V., Chiong M., Criollo A., Moraga F., Garcia A., Härtel S., Jaimovich E., Zorzano A., Hidalgo C. (2008). Changes in mitochondrial dynamics during ceramide-induced cardiomyocyte early apoptosis. Cardiovasc. Res..

[21] Law B.A., Liao X., Moore K.S., Southard A., Roddy P., Ji R., Szulc Z., Bielawska A., Schulze P.C., Cowart L.A. (2018). Lipotoxic very-long-chain ceramides cause mitochondrial dysfunction, oxidative stress, and cell death in cardiomyocytes. FASEB J..

[22] Bielawska A.E., Shapiro J.P., Jiang L., Melkonyan H.S., Piot C., Wolfe C.L., Tomei L.D., Hannun Y.A., Umansky S.R. (1997). Ceramide is involved in triggering of cardiomyocyte apoptosis induced by ischemia and reperfusion. Am. J. Pathol..

[23] Vestri A., Pierucci F., Frati A., Monaco L., Meacci E. (2017). Sphingosine 1-Phosphate Receptors: Do They Have a Therapeutic Potential in Cardiac Fibrosis?. Front. Pharmacol..

[24] Cantalupo A., Gargiulo A., Dautaj E., Liu C., Zhang Y., Hla T., Di Lorenzo A. (2017). S1PR1 (Sphingosine-1-Phosphate Receptor 1) Signaling Regulates Blood Flow and Pressure. Hypertension.

[25] Jin Z.Q., Zhou H.Z., Zhu P., Honbo N., Mochly-Rosen D., Messing R.O., Goetzl E.J., Karliner J.S., Gray M.O. (2002). Cardioprotection mediated by sphingosine-1-phosphate and ganglioside GM-1 in wild-type and PKC epsilon knockout mouse hearts. Am. J. Physiol. Heart Circ. Physiol..

[26] Baranowska-Kuczko M., Kozłowska H., Kloza M., Kusaczuk M., Harasim-Symbor E., Biernacki M., Kasacka I., Malinowska B. (2021). Vasoprotective Endothelial Effects of Chronic Cannabidiol Treatment and Its Influence on the Endocannabinoid System in Rats with Primary and Secondary Hypertension. Pharmaceuticals.

[27] Remiszewski P., Jarocka-Karpowicz I., Biernacki M., Jastrząb A., Schlicker E., Toczek M., Harasim-Symbor E., Pędzińska-Betiuk A., Malinowska B. (2020). Chronic Cannabidiol Administration Fails to Diminish Blood Pressure in Rats with Primary and Secondary Hypertension Despite Its Effects on Cardiac and Plasma Endocannabinoid System, Oxidative Stress and Lipid Metabolism. Int. J. Mol. Sci..

[28] Burstein S., Hunter S.A., Renzulli L. (1984). Stimulation of sphingomyelin hydrolysis by cannabidiol in fibroblasts from a Niemann-Pick patient. Biochem. Biophys. Res. Commun..

[29] Pavoine C., Pecker F. (2009). Sphingomyelinases: Their regulation and roles in cardiovascular pathophysiology. Cardiovasc. Res..

[30] Klevstig M., Ståhlman M., Lundqvist A., Scharin Täng M., Fogelstrand P., Adiels M., Andersson L., Kolesnick R., Jeppsson A., Borén J. (2016). Targeting acid sphingomyelinase reduces cardiac ceramide accumulation in the post-ischemic heart. J. Mol. Cell Cardiol..

[31] Hernandez O.M., Discher D.J., Bishopric N.H., Webster K.A. (2000). Rapid activation of neutral sphingomyelinase by hypoxia-reoxygenation of cardiac myocytes. Circ. Res..

[32] Luiken J.J., Schaap F.G., van Nieuwenhoven F.A., van der Vusse G.J., Bonen A., Glatz J.F. (1999). Cellular fatty acid transport in heart and skeletal muscle as facilitated by proteins. Lipids.

[33] Glatz J.F., Luiken J.J., Bonen A. (2010). Membrane fatty acid transporters as regulators of lipid metabolism: Implications for metabolic disease. Physiol. Rev..

[34] Luiken J.J., Arumugam Y., Bell R.C., Calles-Escandon J., Tandon N.N., Glatz J.F., Bonen A. (2002). Changes in fatty acid transport and transporters are related to the severity of insulin deficiency. Am. J. Physiol. Endocrinol. Metab..

[35] Lago-Fernandez A., Zarzo-Arias S., Jagerovic N., Morales P. (2021). Relevance of Peroxisome Proliferator Activated Receptors in Multitarget Paradigm Associated with the Endocannabinoid System. Int. J. Mol. Sci..

[36] Kalinowska A., Górski J., Harasim E., Harasiuk D., Bonen A., Chabowski A. (2009). Differential effects of chronic, in vivo, PPAR’s stimulation on the myocardial subcellular redistribution of FAT/CD36 and FABPpm. FEBS Lett..

[37] Silvestri C., Paris D., Martella A., Melck D., Guadagnino I., Cawthorne M., Motta A., Di Marzo V. (2015). Two non-psychoactive cannabinoids reduce intracellular lipid levels and inhibit hepatosteatosis. J. Hepatol..

[38] Nagarkatti P., Pandey R., Rieder S.A., Hegde V.L., Nagarkatti M. (2009). Cannabinoids as novel anti-inflammatory drugs. Future Med. Chem..

[39] Devinsky O., Cilio M.R., Cross H., Fernandez-Ruiz J., French J., Hill C., Katz R., Di Marzo V., Jutras-Aswad D., Notcutt W.G. (2014). Cannabidiol: Pharmacology and potential therapeutic role in epilepsy and other neuropsychiatric disorders. Epilepsia.

[40] Watson J.E., Kim J.S., Das A. (2019). Emerging class of omega-3 fatty acid endocannabinoids & their derivatives. Prostaglandins Other Lipid Mediat..

[41] Liu J., Wang L., Harvey-White J., Osei-Hyiaman D., Razdan R., Gong Q., Chan A.C., Zhou Z., Huang B.X., Kim H.Y. (2006). A biosynthetic pathway for anandamide. Proc. Natl. Acad. Sci. USA.

[42] Saghatelian A., Trauger S.A., Want E.J., Hawkins E.G., Siuzdak G., Cravatt B.F. (2004). Assignment of endogenous substrates to enzymes by global metabolite profiling. Biochemistry.

[43] Fu J., Gaetani S., Oveisi F., Lo Verme J., Serrano A., Rodríguez De Fonseca F., Rosengarth A., Luecke H., Di Giacomo B., Tarzia G. (2003). Oleylethanolamide regulates feeding and body weight through activation of the nuclear receptor PPAR-alpha. Nature.

[44] Puhl S.L. (2020). Cannabinoid-sensitive receptors in cardiac physiology and ischaemia. Biochim. Biophys. Acta Mol. Cell Res..

[45] Hiley C.R. (2009). Endocannabinoids and the heart. J. Cardiovasc. Pharmacol..

[46] Bátkai S., Rajesh M., Mukhopadhyay P., Haskó G., Liaudet L., Cravatt B.F., Csiszár A., Ungvári Z., Pacher P. (2007). Decreased age-related cardiac dysfunction, myocardial nitrative stress, inflammatory gene expression, and apoptosis in mice lacking fatty acid amide hydrolase. Am. J. Physiol. Heart Circ. Physiol..

[47] Baranowski M., Zabielski P., Blachnio A., Gorski J. (2008). Effect of exercise duration on ceramide metabolism in the rat heart. Acta Physiol..

[48] Folch J., Lees M., Sloane Stanley G.H. (1957). A simple method for the isolation and purification of total lipides from animal tissues. J. Biol. Chem..

[49] Konstantynowicz-Nowicka K., Harasim E., Baranowski M., Chabowski A. (2015). New evidence for the role of ceramide in the development of hepatic insulin resistance. PLoS ONE.

[50] Gilda J.E., Gomes A.V. (2013). Stain-Free total protein staining is a superior loading control to β-actin for Western blots. Anal. Biochem..

